# Estimated population size of the people who inject drugs in Thai Nguyen, Vietnam: A two survey capture-recapture study using respondent driven sampling

**DOI:** 10.1371/journal.pgph.0000944

**Published:** 2022-12-07

**Authors:** Ly Thuy Nguyen, Anindya K. De, Kim Anh Ai Le, Cuong Manh Pham, Le Khanh, Van Thi Hai Hoang, Abu S. Abdul-Quader

**Affiliations:** 1 Division of Global HIV and TB Prevention, Centers for Disease Control and Prevention, Hanoi, Vietnam; 2 Division of Global HIV and TB Prevention, Centers for Disease Control and Prevention, Atlanta, Georgia, United States of America; 3 HIV department, Provincial Centers for Disease Control and Prevention, Thai Nguyen, Vietnam; 4 Department of Global Health, School of Preventive Medicine and Public Health, Hanoi Medical University, Hanoi, Vietnam; University of Washington, UNITED STATES

## Abstract

To develop an appropriate programmatic response to the concentrated HIV epidemic, program managers require reliable estimates of the sizes of the key populations. This study attempts to estimate the population size of people who inject drugs (PWID) in Thai Nguyen—a province in the northern part of Vietnam. Two source capture-recapture population size estimates were calculated using data from two respondent driven sampling survey rounds conducted in 5 selected districts from May to August 2019. The population size of the PWID was calculated based on the number of PWID recruited in each survey and ‘recaptured’ during the first and the second survey. Additionally, personal network size data collected in the RDS was used to measure the population of PWID using the Successive Sampling Population Size Estimate (SS-PSE) method. The population of PWID estimated in five selected districts using the two capture-recapture method (CRC) (median = 5,396, 95% CI: 4,011–9,100) was slightly lower than estimated using SS-PSE with RDS survey 1 (median = 5,580, 95% CI: 3,024–9,272) and higher than when using SS-PSE with RDS survey 2 (median = 4,793; 95% CI: 2,310–8,618). The provincial PWID population estimates based on various approaches (e.g. extrapolation based on the prevalence of PWID in the districts) ranged from 6,498 (95% CI: 4,829–10,957) to around 6,807 (95% CI: 5,341–10,527). A provincial estimate of 6,782 PWID, with a confidence interval ranging from 5,312 to 10,527, will help guide planning and resource allocation to support appropriate levels of HIV prevention, care, and treatment services in the Thai Nguyen province.

## Introductions

It is estimated approximately 230,000 adults were living with HIV in Vietnam in 2020 [1], of whom more than 170,000 people are receiving antiretroviral therapy [2]. In 2020, among people who inject drug, estimated national HIV prevalence was 12.7% and antiretroviral therapy coverage for those who are HIV positive was only 64% [2]. Despite the significant burden of HIV in Vietnam and the role PWID play in the HIV epidemic, available population size estimates (PSE) have substantial limitations. Moreover, most estimates are outdated, focusing on big cities, such as Ho Chi Minh City, and most likely do not reflect Vietnam’s ever-expanding key population sizes. Updated PWID size estimates will help national and provincial authorities and other stakeholders understand continuing epidemic patterns and will inform and enable disease control and prevention efforts, HIV programmatic coverage, and evaluations.

In Vietnam, drug use is highly stigmatized and widely perceived as a severe social problem rather than a public health issue. Law enforcement has taken a very strict approach sending PWIDs to compulsory treatment centers for crime prevention [3]. The view on the use of drugs has led to an underground drug user community, offering limited data on the number of drug users. Previous estimates of PWID in other provinces in Vietnam have successfully used capture-recapture with respondent-driven sampling (RDS) [4, 5] demonstrating that the network based chain referral methods can be used to recruit hidden populations in provinces where drug use is stigmatized and policed.

This paper describes the implementation of and findings from a population size estimation activity among PWIDs in Thai Nguyen. Thai Nguyen is one of the seven provinces supported by the US Centers for Disease Control and Prevention (US.CDC)–Vietnam. The intensive support provided by US.CDC–Vietnam focuses on achieving the Joint United Nations Programme on HIV/AIDS (UNAIDS) 90-90-90 targets which include 90% of HIV-positive persons to be diagnosed, 90% of those diagnosed with HIV will receive sustained antiretroviral therapy, and 90% of those receiving antiretroviral therapy will achieve viral suppression by 2030 [6].

In Thai Nguyen, there were an estimated 6,000 people living with HIV (PLHIV) in 2020 [1]. Of these, about 92% of them know their status. Among people knowing their HIV status, approximately 80% were on antiretroviral therapy (ART) [7]. PWID in Thai Nguyen continue to account for a significant proportion (55.7%) of the overall PLHIV in the province [8]. Data from the most recent HIV sentinel surveillance (2019) indicates an HIV prevalence of 9.1% with 95% Confidence Interval (95% CI): 6.3%-13.0% among PWID in Thai Nguyen [9]. Provincial PSE of PWID were 5,007 in 2018 based on the national program reporting system (circular 03) [7]. However, no information is available on the detailed characteristics of the targeted population (e.g injecting behaviors, injecting duration, age, gender) and method(s) used to arrive at such an estimate. Moreover, stigmatization may keep PWID from utilizing program services. To understand the extent of the provincial epidemic and the need for programmatic response, better data on population size estimation of PWID is needed. This information is key for acquiring more precise estimates of the PLHIV, in measuring coverage of various prevention and care interventions, and in designing adequate HIV programs and services.

## Methods

Using respondent-driven sampling (RDS) [10], we implemented a two-source capture-recapture method to estimate the population size of PWID in Thai Nguyen. Study populations included men and women aged 18 years or older who lived in the province for the past six months and reported injecting illicit drugs (not prescribed for treatment purposes) within the previous three months. RDS is a type of chain referral sampling employing a dual system of compensation [10]. RDS draws on both Markov-chain theory and the theory of biased networks [10] and can reduce the biases generally associated with other chain referral methods. Even though sampling begins with an arbitrarily chosen set of initial subjects (the seeds), the composition of the ultimate sample is independent of those initial non-randomly selected subjects. Implementation involved the following steps: (1) Selection of the districts where data were collected; (2) Implementation of the first RDS survey (the capture survey); and (3) Implementation of the second RDS survey (the recapture survey) in each selected districts (4).

### Selection of districts

In Thai Nguyen, nine administrative districts cover approximately 3,563 square kilometers with a total population of 1,255,070 [11]. According to the Public Security Office, about 5,202 people in the province used drugs in 2018 [12]. The data, however, does not distinguish between injectors and non-injectors nor between current and former injectors.

The Public Security Office also breaks down the reported number of drug users by the district. The proportion of drug users in Thai Nguyen for each district were calculated using the public security numbers in 2018 with underlying assumption that the distribution of PWID across districts was the same as all drug users Five districts were selected for the size estimation activity based on the consideration of access accounting for 79% of the police-estimated number of drug users in Thai Nguyen. Six study sites were established for data collection, with one study site in 5 each of the 4 selected districts and two study sites in Thai Nguyen city which was the largest district within Thai Nguyen province (Table 1). The selection of study sites was based on discussions with the provincial and district authorities to ensure the sites were accessible to the participants and discreet enough to not attract attention.

**Table 1:** Weighted PS estimate (CRC) in survey districts.

District	Round 1 n	Round 2 n	Recapture n	Naïve PSE	Round 2 Wtd n	Recapture Wtd n	Weighted PSE	LL PSE	Median PSE	UL PSE
**TN city**	263	263	69	2022	2042	260	1900	1238	1953	4666
**Dai Tu**	130	130	12	1408	1400	95	1914	1120	1927	4452
**Pho Yen**	130	128	47	354	326	95	447	342	449	618
**Phu Luong**	130	130	79	214	214	122	227	196	227	268
**Phu Binh**	130	130	28	604	600	130	599	419	603	999
**All (5 survey districts)**							5087	4143	5396	9118

* LL: lower limit. UL: upper limit.

### RDS capture-recapture survey

#### Sample size

The sample size for the study was based on the estimation of population size using the capture-recapture method. It was calculated using normal approximation to hypergeometric distribution following the approach of Robson [13]. Based on the precision level of PSE and available resources, the sample size for each round of RDS was estimated to be 783, allowing for a design effect of 1.7. Sample sizes of 263 in Thai Nguyen city (TN city) (2 survey sites) and 130 in each of the 4 districts (4 survey sites) were planned to reach the total sample size of 783 for the study in each round of RDS.

#### Sampling strategy

After selecting the 6 study sites, data including eligibility assessments and individual interviews were collected. RDS, including coupons with unique numbers specific to each study site, was used for recruiting participants. Each coupon contained information such as codes, study site locations, telephone numbers, hours of operation, and valid recruitment dates.

For each survey round, local community-based organizations recruited six different PWID to serve as survey initiates at each site. These initiates or “seeds” were selected to reflect variation in characteristics, for example, age, residential area, length of time as PWID, and methadone maintenance treatment (MMT) status. After completing the eligibility assessment and questionnaire, each seed was given three referral coupons to distribute to their peers and to encourage them to participate in the study. The peers who visited the study sites completed the same eligibility assessment and questionnaire as the seeds. The peers subsequently received three recruitment coupons and were asked to distribute their coupons to their peers. Study participants were guided to ask their peers to come to the study site where their recruiters originated from.

Participants will receive compensation for their participation and for each successful referral (up to a maximum of three) in the survey. Each round of recruitment continued for about 4 to 5 weeks until the study site reached the target sample size. Three weeks after completing the first (capture) survey, an independent second (recapture) survey, similar to the first, was conducted in the 6 selected study sites. The assumption of capture–recapture method requires the two samples of two surveys are independent. In other words, the probability of sample selection in the first survey (capture) does not affect the probability of sample selection in the second survey (recapture). Therefore, the seeds selected in the second survey were people who did not participate in the first survey.

#### Eligibility criteria for the participants

The target populations included men and women aged 18 years or older who reported injecting illicit drugs (not prescribed for treatment purposes) within the previous three months and who lived in Thai Nguyen for the last six months. After completing the eligibility assessment and obtaining verbal consent, participants were assessed using a structured questionnaire. In Vietnam, injecting drug is illegal and obtaining written consent can prevent the participants from participating in the study. As such, we requested a waiver of documentation of informed consent. No name, individually identifiable information, or signature of participants were put in the consent form. This is to gain trust from the participants to the survey staff and to ensure complete anonymity of their participation.

#### Assessment at study sites

Brief interviews lasting 15 to 20 minutes were conducted. Interview questions included basic demographics, network size, length of drug injection, and their use of MMT facilities. Individuals who came to a study site without a valid coupon were not assessed or interviewed.

#### Fingerprint matching

Even though we had multiple study sites to cover all five selected districts, two selected districts may be next to each other, and given the nature of the PWID networks, potential participants may receive more than one coupon and may attempt to enroll in the survey multiple times. Coded fingerprint biometric authentication was used to avoid potential duplications (one person participating in the same survey twice) and to determine how many eligible participants were new captures and how many were recaptures [4]. If the codes generated during the second survey matched those generated during the first survey, they were considered recaptures. The coded fingerprint biometric could not be decoded to identify any individual participant personally.

#### Population size estimates of the sampled districts

Since RDS is not an equal probability sampling approach, in addition to naïve (unweighted) estimate, weighted estimates for the capture-recapture samples were calculated [14].

The formulas used for the two estimates included the following:

$$N_{naïve}=\frac{(\sum_{k}I_{k}^{1,0}+\sum_{k}I_{k}^{1,1})(\sum_{k}I_{k}^{1,1}+\sum_{k}I_{k}^{0,1})}{\sum_{k}I_{k}^{1,1}}$$

$$N_{wtd}=\frac{(\sum_{k}I_{k}^{1,0}+\sum_{k}I_{k}^{1,1})(\sum_{k}\frac{I_{k}^{1,1}}{\pi_{k}}+\sum_{k}\frac{I_{k}^{0,1}}{\pi_{k}})}{\sum_{k}\frac{I_{k}^{1,1}}{\pi_{k}}}$$

$$Var(N_{naïve})=N_{naïve}\times \frac{\left(\sum_{k}I_{k}^{1,0})(\sum_{k}I_{k}^{0,1}\right)}{{\left(\sum_{k}I_{k}^{1,1}\right)}^{2}}$$

The summation below is in reference to all study participants: *π*_*k*_ probability of inclusion of the *k*th individual; in $I_{k}^{j,l}$, *j* and *l* stand for the first and second samples respectively and value is one or zero to indicate presence or absence a participant, and $I_{k}^{j,l}$ takes the value one if the status of individual k stratifies sample status indicators *j* and *l* and is zero otherwise. The inclusion probability for RDS depends on the network size and the recruitment process, and its derivation is based on Markov chain theory and biased network theory. Given the necessary inputs, software such as RDS-A [15] can generate the weights which are reciprocal of the inclusion probabilities.

The confidence interval (95% CI) for the naïve estimate was calculated as $N_{naïve}\pm 1.96\sqrt{Var}$. The confidence interval for the weighted estimate was calculated using bootstrap simulations. This involved drawing samples repeatedly with replacement from the RDS sample with marked recapture, and weighted estimates were generated for each repetition. The set of estimates thus generated formed a simulated sample of PSEs. The median estimate of the resampled estimates is the estimated median of the district population size and the upper and lower confidence limits were determined using the 97.5^th^ and 2.5^th^ percentiles of the simulated PSEs.

In addition, successive sampling population size estimates (SS-PSE) for RDS surveys [12] were also derived for each site and round. Basic RDS information like recruitment date, coupon codes, network size, and mid-value of the PSE, which was set equal to the median value from the simulation of weighted estimates, as described earlier were provided. We calculated and used implicit visibility for network size and finally incorporated quartile (Q2 or Q1 and Q3) values for PSE, which came from simulated samples of PSEs. Imputed visibility is a method to adjust weights via adjusted network size and it is based on a measurement error model. This attempts to adjust the correct self-reported network sizes which are prone to various types of some biases and errors [16]. Analyses were conducted using software RDS-A and Stata 15 [15, 17]. All participants including seeds are included in all analyses. Estimates of the demographic characteristics of the PWID population for each round were calculated using Gile successive sampling estimator and results of the PWID population size estimates of the current study.

#### Estimation of PWID population size for the whole province

Median estimates of the PWID population size, based on simulations for each surveyed district, were used with the general population estimates or data to derive the estimated PWIDs per 1000 persons. The median of this ratio was then used to estimate the PWID population for the rest of the districts which were not included in the study sample. The same procedure was followed using police and program report data in place of the survey estimates to compare the results based on information from sources external to the study. This process was repeated with the male population as the base to check for consistency and external validation.

A simple approach of proportionately inflating the estimate of the total five districts surveyed by a factor was also used. According to the program or police reports, the proportionality factor was determined based on the percentage of PWIDs in the districts covered by the survey compared to the whole province. Using the police and program estimates as external validations is important as these estimates are usually used for HIV program planning when actual estimate exercise is not available. Thus, we want to compare these estimates with the present study estimate to identify the gaps and provide recommendations for HIV resource allocation.

Three parametric models were used to calculate the confidence limits of the estimates of districts that are not in the sample. The first model was based on the Poisson distribution, the second model on the lognormal distribution, and the third model on the negative binomial distribution (NGB). Model parameters are estimated first utilizing the estimated population size for the Poisson model which has one parameter which is also the mean of the distribution. For the lognormal, there are two parameters and so estimated PSE and median value of the standard deviation of the log transformed simulated PSEs of the 5 survey districts were used. Finally, for negative binomial, the two parameters were derived from the PSE and the variance of the simulated PSEs of the survey districts. Since the variance of NGB varies proportionally to the mean, variance of the survey district with PSE close to that of the non-survey district with the largest PSE was used. Once the models are fully identified, simulated samples were generated to calculate the lower (2.5^th^ percentile) and upper limits (97.5^th^ percentile) of the confidence interval. The confidence limits for the province total were then calculated from the simulated estimates under three models for the non-sampled districts combined with the resampled estimates of the survey districts.

#### Human subject approval

The study protocol received ethical approval from the Institutional Review Board of the Hanoi Medical University, Vietnam (Approval number 13/HMUIRB). The protocol was also reviewed per the Centres for Disease Control and Prevention (US CDC) human research protection procedures and was determined to be research but CDC was non-engaged (HSR tracking number: 2019–165). CDC investigators did not interact with human subjects or have access to identifiable data or specimens for research purposes.

## Results

### Characteristics of participants

There were 783 participants recruited in the first survey round and 781 participants recruited in the second survey round in six survey sites. The successful rate of having three coupons given out by a recruiter returned by the recruits were 25% for round one and 32% for round two. The overwhelming majority of participants were male (98.8%—round one and 99.4%—round two), married (63.5%—round one and 61.1%—round two), with mean age ranging from 42.0 years in round one to 41.9 years in round two. More than three-third of PWID reported low educational levels at primary and secondary schools (72.3%—round one and 74.3%—round two). The average duration of drug injection was 13.3 years for round one and 11.9 years for round two. When asked about MMT, 68.9% (CI: 63.9–73.8) of PWID in round one and 68.1% (CI: 61.7–74.3) of PWID in round two indicated they have ever been on methadone. And 58.8% (CI: 52.7–64.9) of PWID in round one and 59.7% (CI: 53.8–65.6) of PWID in round two indicated they were on methadone in the past month.

### Estimates of PWID population size

#### Weighted estimates of PWID population size of survey districts using capture-recapture method

Weighted estimates, which are appropriate in the RDS context given the unequal selection probabilities, are given in Table 1. Median estimates based on simulation are also shown along with the 95% CIs. Weighted estimates are similar to the corresponding median estimates. The CIs from the simulation indicate the PSE distributions are positively skewed. According to the naïve estimates, TN city has the largest number of PWIDs (2022), followed by Dai Tu (1408). However, the weighted estimates are similar in the two districts (median estimates TN city- 1953 and Dai Tu- 1927). Except for the PSE of Dai Tu, naïve and weighted estimates for the rest of the districts are not very different.

It may be noted that the median estimate of the 5 districts was 5,396 and was higher than the sum of the median sizes possibly because of the right skewness of the distributions. The weighted estimated CI of the five districts was much wider (about double) than the unweighted CI (not shown) reflective of the effect of unequal RDS survey weights. Unlike the naïve CI estimate, the weighted CI was not symmetric. This pattern is also true for individual district CIs, which are 1.65 to 2.3 times wider than the corresponding naïve estimate CIs.

#### Estimates of PWID population size of survey districts using Successive Sampling estimates (SS-PSE) method

Two sets of SS-PSE were computed, i) only median information for the PSE prior, and ii) first and third quartile (Q1 and Q3) information for the PSE prior were provided in addition to the median. Choice of Q1 and Q3 were guided by the simulation distribution for the weighted estimate. The findings of this study suggest, providing Q1 and Q3, the first and third quartiles produced more consistent results than those based on Q2 alone. Thus SS-PSE estimates for which Q1 and Q3 were specified for the priors are presented (Table 2), and it may be noted the same values for Q1 and Q3 were provided for both round 1 and round 2 calculations. Population size estimates and CIs for the two rounds were very close, for all the districts except for Dai Tu, where the round 2 estimate was 32% lower than round 1 (1,681 vs 2,467). This difference resulted in a 14% difference in the 5-district total (4,793 vs 5,580). SS-PSE results were comparable but slightly lower than the weighted estimates for most districts except for round 1, SS-PSE for Dai Tu, which was 28% higher. However, the weighted median estimate (5,396) fell in between the SS-PSEs from the two rounds and was comparable and was used in subsequent calculations.

**Table 2:** Successive sampling PSE (SS-PSE) in survey districts.

District	Mean		Median		95% CI			
	Round 1	Round 2	Round 1	Round 2	Round 1		Round 2	
**TN city**	1829	1939	1695	1814	856	3560	879	3713
**Dai Tu**	2640	1807	2467	1681	877	5191	734	3656
**Pho Yen**	447	447	440	437	339	594	331	618
**Phu Luong**	227	225	223	222	189	281	186	279
**Phu Binh**	573	560	557	549	371	864	338	833
**All (5 districts)**			5580	4793	3024	9272	2310	8618

#### Estimates of PWID population size for the Thai Nguyen province

Using median weighted estimates, PWID prevalence (number of PWID per 100 general population members) was highest in Dai Tu at 1.178% and was followed by TN city with 0.592%, Phu Binh (0.431%), Pho Yen (0.331%), and Phu Luong (0.212%). The provincial PWID prevalence was therefore resulting in a median of 0.431 (Table 3). Using program and police data, median values were 0.427 and 0.440, respectively, similar to that based on weighted estimates. The median among these (prevalence = 0.431) was used to calculate the estimates for the districts not covered in the survey. Thus, the total PWID population size for the province was estimated to be 6,498. If the higher value of 0.440 were used, the estimate for the province would have been 6,524. Following a similar approach but using the male population instead of the general population in the districts, the estimate was 6,431. Using a simpler approach with the proportion of PWIDs covered by the surveyed districts and using the average of program and police data then extrapolating for all the districts, produced an estimate of 6,858 for the province.

**Table 3:** Surveyed districts and PS estimates for all 9 districts (CRC).

Districts	General Pop	Police count	Police based % prevalence	Program count	Program based % prevalence	Weighted PSE (CRC)	Weighted estimate % prevalence
**TN city** *	330000	1939	0.588	1939	0.588	1953	0.592
**Pho Yen** *	135711	726	0.535	726	0.535	449	0.331
**Dai Tu** *	163637	698	0.427	720	0.44	1927	1.178
**Phu Binh** *	139753	358	0.256	358	0.256	603	0.431
**Phu Luong** *	106834	432	0.404	465	0.435	227	0.212
**Dong Hy**	107769	426		426		465	
**Song Cong**	49983	242		302		216	
**Dinh Hoa**	90086	208		850		389	
**Vo Nhai**	62326	173		173		269	
**Median or Total**			0.427		0.44	6498	0.431

*5 districts where survey site were established.

#### Variability assessment of population size estimates

Assessment of variability in the districts outside the survey area and for the overall Thai Nguyen province was conducted, using Poisson, Lognormal, and Negative Binomial distributions. Variance estimates for the models were based on the variances of simulated data for surveyed districts with similar PSEs. The Poisson model produced very narrow 2.5^th^– 97.5^th^ percentile intervals and was not considered useful (not presented). In contrast, Lognormal and Negative Binomial models produced more realistic intervals, that agreed reasonably well with the variability of the simulated data for the surveyed districts (Table 4). Lognormal-based lower limits were a good fit, but upper limits fell short for the larger PSEs for TN city, and Dai Tu. The Negative Binomial-based upper limits fitted well, but lower limits were overshot for the two larger PSEs. Estimates for the whole province were similar for both models. Medians were 6,807 vs. 6,757, and the same was true for interval estimates. The median of these two estimates is 6,782 with CI: 5,312 (= min of LLs) to 10,527 (= max of ULs), can serve as the point PSE for the province. These model-based median PSEs were similar to the simple proportional estimate but larger than the weighted estimate based on program and police data.

**Table 4:** Modeled CI estimates.

Districts	Lognormal				Negative binomial			
	Mean	Median	LL	UL	Mean	Median	LL	UL
**Song Cong**	221	216	140	335	216	208	97	376
**Dinh Hoa**	399	387	253	598	389	385	261	539
**Dong Hy**	478	467	302	725	465	462	337	617
**Vo Nhai**	275	268	175	411	268	261	148	424
**Thai Nguyen province** *	7103	6807	5341	10527	7066	6757	5312	10513

* Based on simulation for 5 surveyed districts and models for 4 districts outside the survey. LL: lower limit. UL: upper limit

## Discussion

This was the first study to estimate the population size of the PWID in Thai Nguyen province, Vietnam. A two-sample capture recapture design using RDS and successive sampling population size estimates (SS-PSE) were used in the 5 selected districts. The mean age of PWID in Thai Nguyen was more than 40 years, higher than the 36.7 years among PWID participating in HIV national sentinel surveillance 2019 in 15 provinces [9] but close to Hai Phong PWID mean of 39 years in 2018 [5]. The difference may reflect the aging population of PWID in Thai Nguyen or the PWID population members being present in Thai Nguyen earlier than in other provinces. The mean years injecting in 2013 HIV/STI Integrated Biological and Behavioral surveillance ranged from 5.6 to 8.2 years in 9 provinces [18], compared to 11.9–13.3 years in our study.

### Estimates of PWID population size using capture-recapture estimates method

Based on various statistical models, all approaches produced similar population size point estimates for PWIDs in the Thai Nguyen province. The largest estimate (lognormal model- 6807) differed from the smallest (negative binomial model-6757) by 50. This is reassuring as it served as a validation of the results. Confidence intervals of the estimates were also comparable. One main reason for this is that a significant portion of the PWID were covered in the surveyed districts. The contribution of the modeled estimates of the four remaining districts was minimal. It is interesting to note the simple proportional estimate was also close for these estimates.

We looked at the model fit by comparing the simulated CIs with the model-generated CIs among the five districts in the sample. Poisson model did not fit well for the districts with large PWID but fit was satisfactory for PWID estimated at 400 or less. Since the districts outside the survey had a small PWID population, we decided to include them as contenders. Lognormal models produced a better fit. The model closely matched all lower limits but, the upper limits of the two districts with the largest PWID fell short. Negative binomial models produced better upper limits, but the lower limits for the districts with large PWID were much smaller than those based on simulation. These right skewed distributions were selected instead of a symmetric distribution like normal because the population size distribution is characteristically asymmetric with a skew to the right. This was supported by the simulated CIs for the weighted estimates and that of SS-PSEs.

### Estimates of PWID population size using successive Sampling estimates (SS-PSE) method

While the advantage of SS-PSE was that it requires only one RDS survey, the quality of the SS-PSEs depended on good prior information. Researchers have noted effect of violation of important assumptions for SS-PSE including how sensitive the estimates can be based on the prior especially when the sample size is small compared to the population size (<1/10) [19, 20]. Without prior information, results were not stable and varied widely. More information on prior, like Q1 and Q3, in addition to the median led to estimates similar to the weighted capture-recapture (CRC) (+13%) except for Dai Tu round one. In Dai Tu, a large difference was found between mean network (with high SD) and imputed visibility size for round one data. This might indicate network sizes and thus weights were not reliable in that round. It may also be noted that naïve and weighted CRC estimates differed substantially for Dai Tu. The round two estimate is in line with the weighted estimate and seems more reliable.

The Ho Chi Minh PWID study [4] reported RDS based PSEs for 7 districts. The asymmetry reflected in the CIs shows that the differences between upper limits and medians are about 1.75 times than that between corresponding medians and lower limits (range 1.6 to 3.1, median 1.75). The CIs for SS-PSEs in Thai Nguyen districts showed a similar pattern and the factor was around 1.7 (range 1.3 to 2.3 and median was 1.7).

### Estimates of PWID population size using Police/program estimates versus study estimates

Historically, PWID in Vietnam are heavily policed and broadly labeled as “criminal” or “evil” [3]. However, Thai Nguyen, along with other PEPFAR supported provinces, has generally led the movement towards evidence-based substance use treatment and HIV prevention in Vietnam. This includes the involvement of multiple evidence-based interventions, including syringe access programs, antiretroviral treatment for HIV infection, and MMT (10 clinics as of December 2019). Drug users can voluntarily receive MMT in clinics or they will receive compulsory treatment at “06” center. For most districts, the estimates from the two sources are similar except for Dinh Hoa. Police and Program authorities might consider validating and reconciling the estimates and provide reasons for any significant differences. This is understandable as the estimates on this study are higher than program/police estimates, which are only based on the number of drug users accessing program services and are in the compulsory treatment centers or are in the methadone clinics. The underestimation of the PWID population may lead to under-allocation of resources for prevention and care that may limit access to services. In the last decade, the perception of injecting drugs has gradually shifted from a social problem to a public health matter. The updated size estimation of the hidden population of PWID in this study will inform service planners to move forward with improved and appropriate HIV prevention programs. Especially in the current context, when the rate of HIV infection among PWID in Vietnam is decreasing, Thai Nguyen is one of eight provinces where the epidemic is rising alarmingly.

### Limitations

The estimates of PWID population size did not include those younger than 18 years and those who were very mobile. Those participating in this study represent a subgroup of the PWID population in Thai Nguyen, accessible to the study team. Among six study sites, three sites were close to MMT clinics, which may have led to the oversampling of participants on methadone in this study. Moreover, although efforts were made to ensure only actively eligible drug injectors were recruited through careful screening for injection marks, a few non-active drug injectors may have enrolled in the study.

When participants reported their network size, it is possible that they might have included people from other districts. We believe however, that this is not common because PWID networks are mostly confined in small geographic localities. The simulation-based CIs for weighted median PSEs did account for the unequal inclusion probabilities but not for the dependency among the sample units this might have led to narrower CIs. However, the widths of CIs are similar to those of SS-PSEs (+/- ~20%) for most which suggest that the dependency effect might be small.

While these are important limitations, the authors believe they do not negate the strengths of the study, which included the application of rigorous dual capture and recapture methods to assess the consistency of PWID population size estimates.

## Conclusion

An estimate of approximately 6,782 injectors, with a confidence interval ranging from 5,312 to 10,527, provides a sound basis for resource allocation decisions to reduce HIV transmission and to provide HIV care for the PWID population in Thai Nguyen. The two-source capture and recapture population size estimates provide greater confidence in the results, which provided a more reliable estimate of PLHIV in the province and a better understanding of where new infections might be occurring with increased frequency. The findings may be helpful in governmental, civil society, and clinical settings in guiding planning and the allocation of resources for an HIV strategy designed to end the HIV epidemic in the province.

## Supporting information

S1 Data July 2020 data. (XLSX)

S1 Questionnaire Inclusivity in global research questionnaire. (DOCX)
